# Supplementary material for: Autoregulation of the LIM kinases by their PDZ domain
Source: Nat Commun. 2023 Dec 19;14:8441. doi: 10.1038/s41467-023-44148-4 (PMC10730565; doi:10.1038/s41467-023-44148-4)
Supplement: Supplementary file 1 — Supplementary Information [file 41467_2023_44148_MOESM1_ESM.pdf]

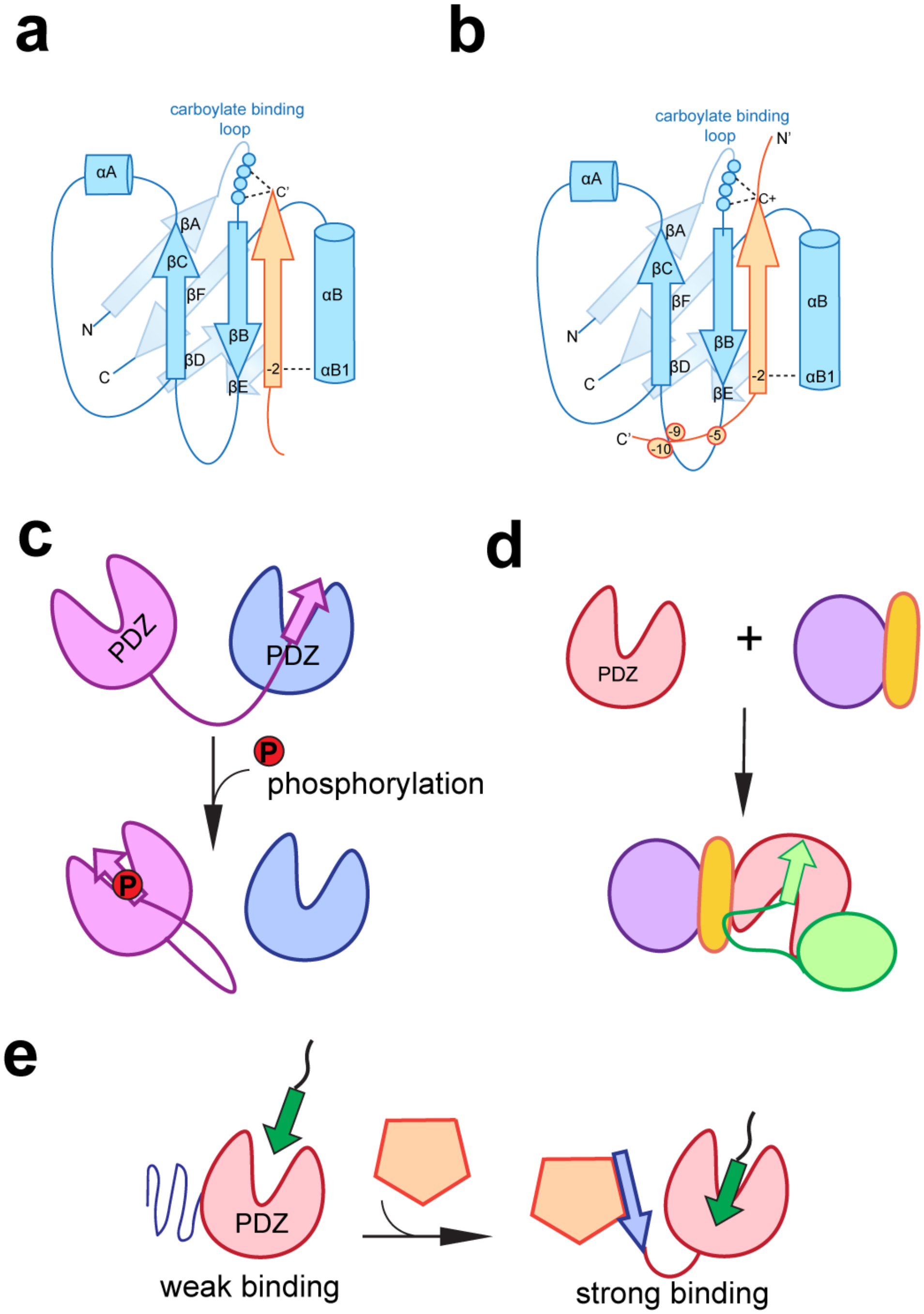

Supplementary Figure 1. Canonical and non-canonical PDZ domains. a) Schematic cartoon illustrating binding of a canonical PDZ domain to a C-terminal peptide. b-e) Non-canonical PDZ domain interactions illustrated in cartoon format, including peptide binding to internal and extension motifs (b) regulation by phosphorylation (c), noncanonical interaction surfaces (d), and allosterically induced conformational changes that alter binding partner interactions (e).

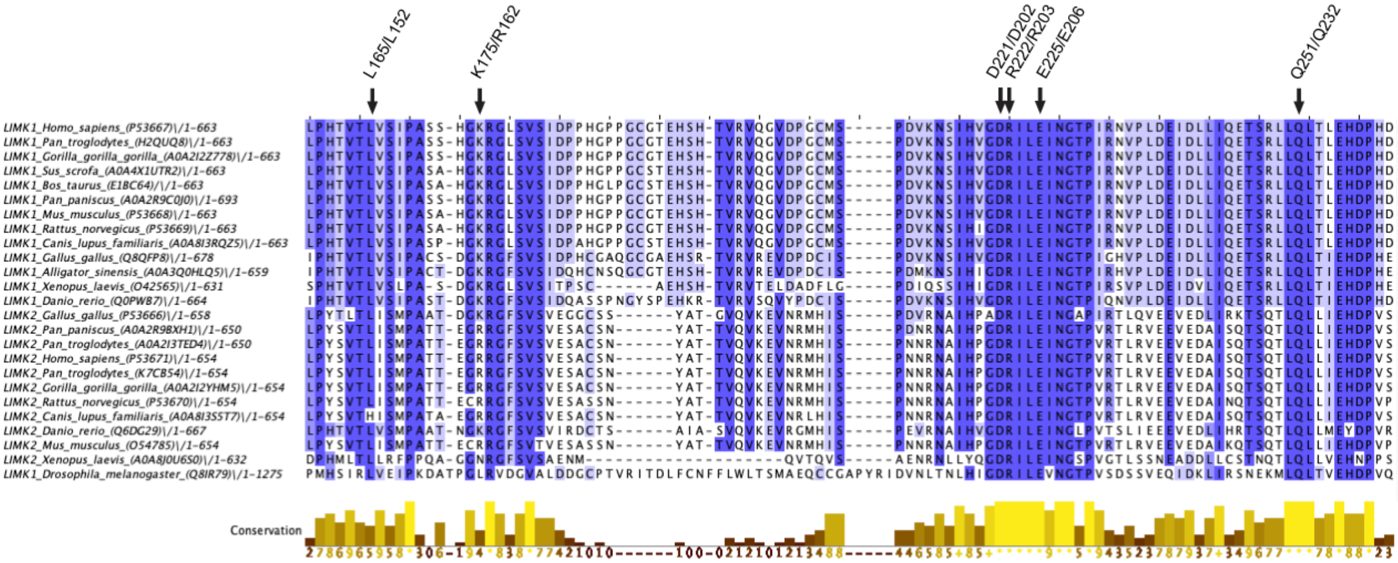

Supplementary Figure 2. Conservation of the LIMK PDZ domain. Sequences were obtained from UniProt[78] and aligned in ClustalOmega[71]. Each sequence is named by species and UniProt ID. Conservation scores calculated in Jalview[72]. Identical residues are highlighted in dark blue, and partially conserved residues in light blue. Mutations used in kinase assays are shown under an arrow and their LIMK1/LIMK2 residue numbers shown.

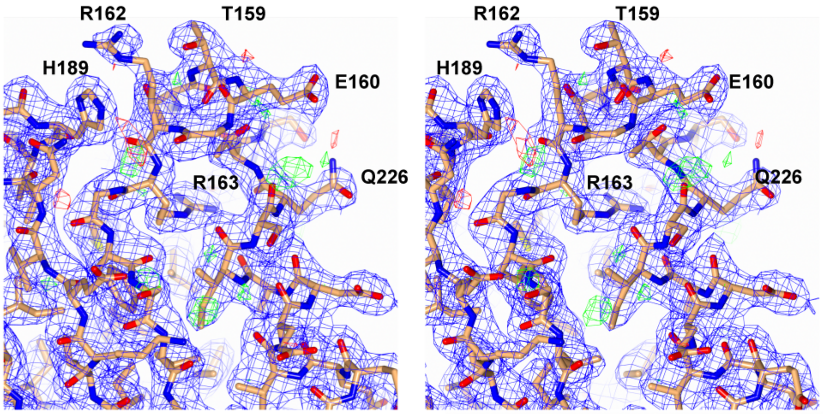

Supplementary Figure 3. Representative electron density of LIMK2 PDZ domain. Stereoview of final refined electron density map for LIMK2 PDZ domain. $2F_{obs} - F_{calc}$ electron density map contoured at 1σ (blue). $F_{obs} - F_{calc}$ electron density map contoured at +3σ (green) and -3σ (red). Image generated using CCP4mg[77].

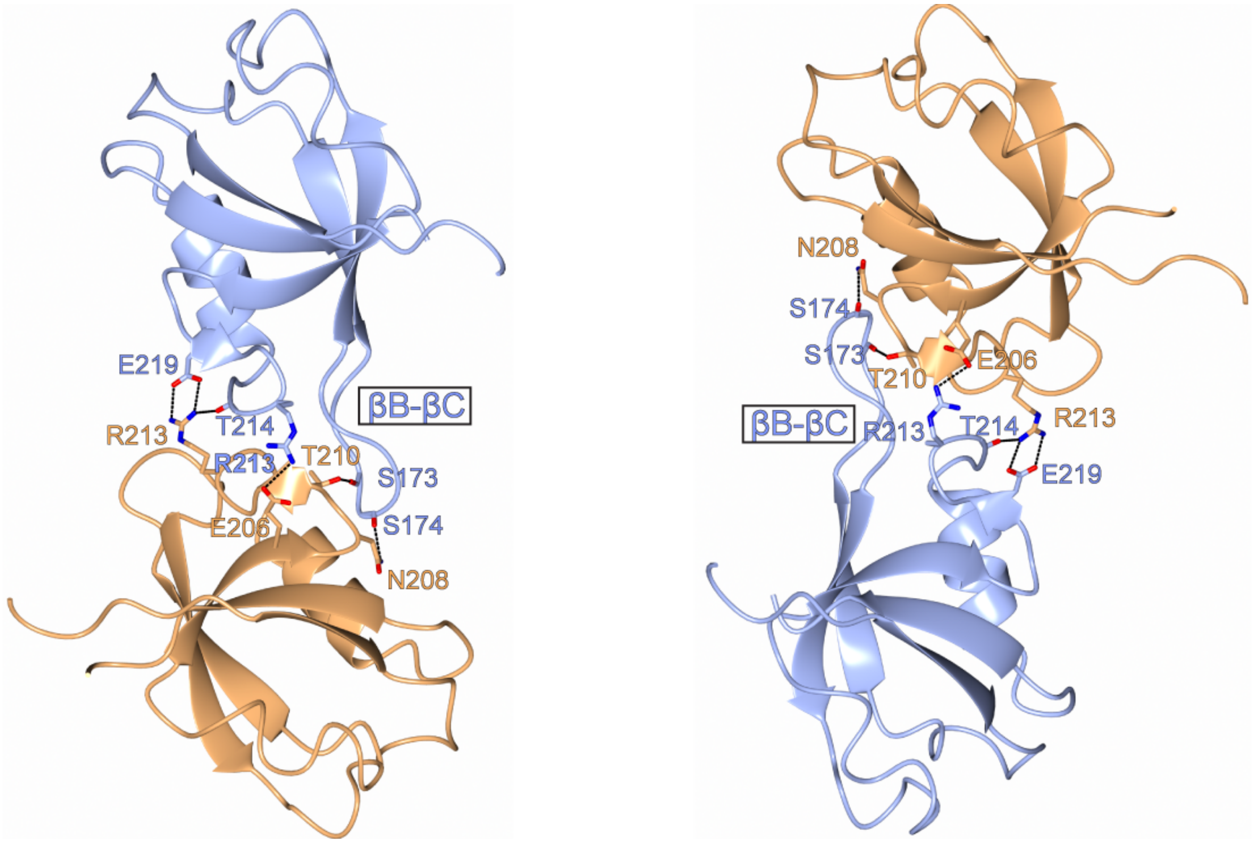

Supplementary Figure 4. Comparison of two determined orientations in the crystal. The structure contains 8 copies of the PDZ domain (termed copies A-H) per asymmetric unit. All 8 copies exhibit good electron density and superpose with a root-mean-square deviation (RMSD) of 0.3±0.1 Å over 89 equivalent Cα positions. Superposition of all 8 asymmetric unit members reveals a variation in the conformation of the βB-βC loop in copies A, C, D, H compared to copies B, E, F, G. Hydrogen bonds between conformations are indicated, along with the βB-βC loop. Image generated using CCP4mg[77].

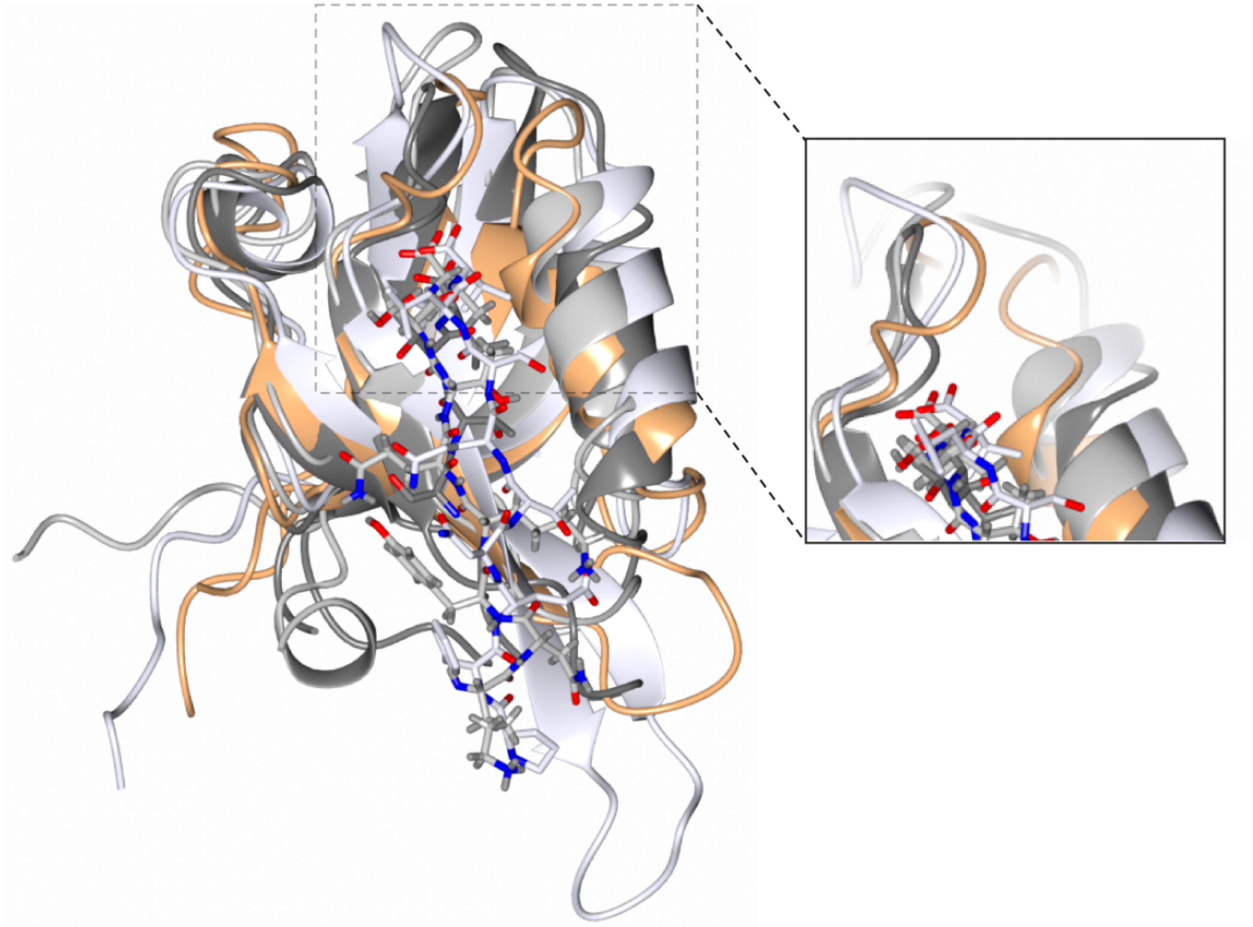

Supplementary Figure 5. Canonical vs LIMK PDZ binding. Comparison of the αA-βF loop orientation of LIMK2 PDZ crystal structure (orange) and the most similar PDZ domains structures as designated by Dali search[76]; membrane-associated guanylate kinase with inverted domain structure protein 1 (MAGI-1) PDZ2 bound to RSK1 peptide, PDB ID: 5N7D[79] (dark grey), The third PDZ domain from the synaptic protein PSD-95in complex with a C-terminal peptide derived from CRIPT, PDB ID: 5HEY[52] (grey), PDZ domain from Human microtubule-associated serine/threonine-protein kinase 1(MAST1) in complex with a class I C-terminal peptide sequence, PDB ID: 3PS4 (light grey). Images generated using CCP4mg[77].

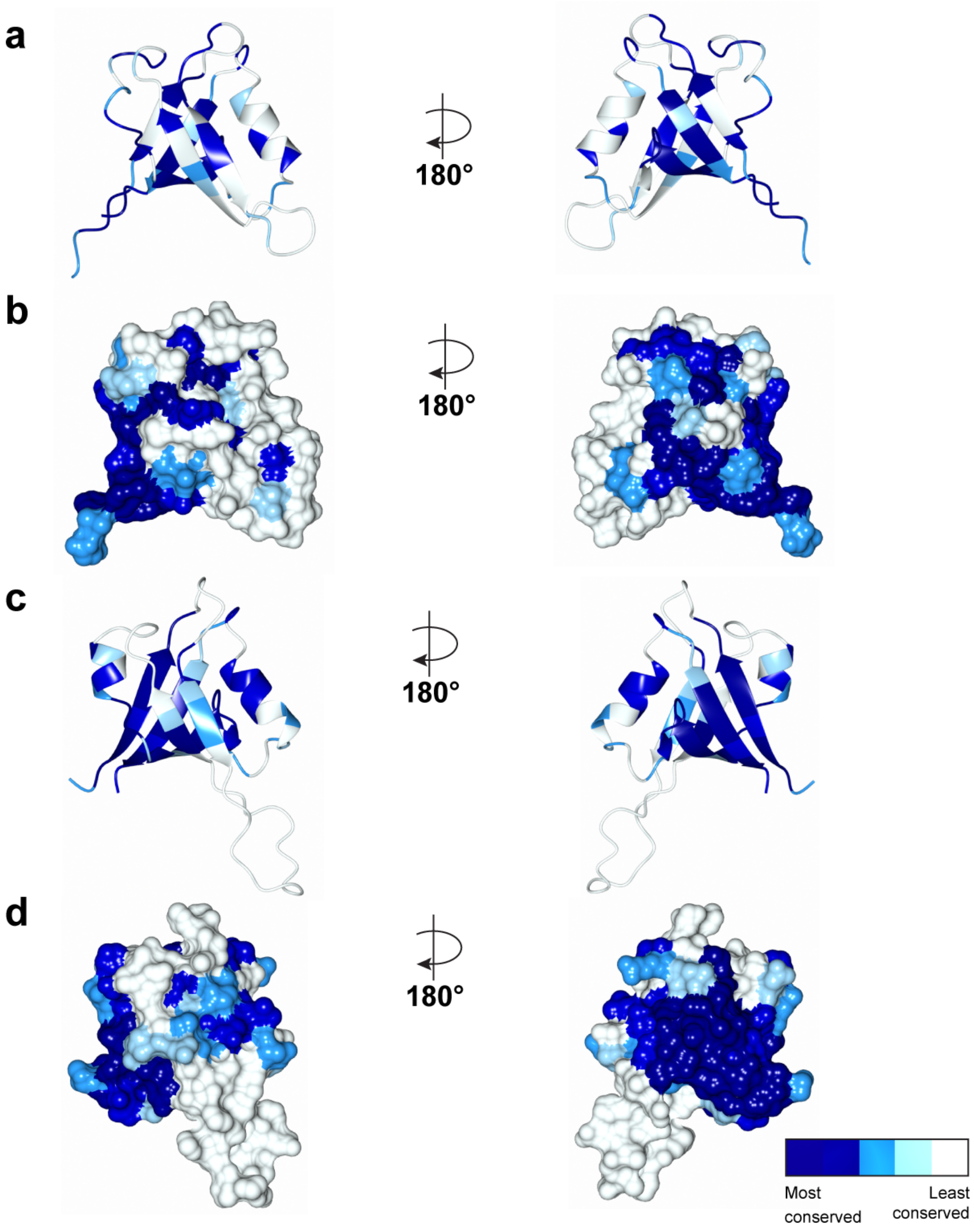

Supplementary Figure 6: Conservation of the PDZ domain within LIMK2 and LIMK1 sequences. a, b) Cartoon and surface representations showing conservation of the LIMK2 PDZ domain across LIMK2 sequences mapped to our crystal structure. Sequence alignment of 209 sequences of LIMK2 was made using Clustal Omega[71]. Species in this alignment include mammals, birds, fish, and insects. c, d) Cartoon and surface representations showing conservation of the LIMK1 PDZ domain across LIMK1 sequences mapped to residues 160 to 260 of the AlphaFold model (AF-P53667-F1-model_v2). Sequence alignment of 212 sequences of

LIMK1 was made using Clustal Omega[71]. Completely conserved residues are colored dark blue, less strongly conserved residues colored lighter shades of blue, and non-conserved residues in white. Image generated using CCP4mg[77].

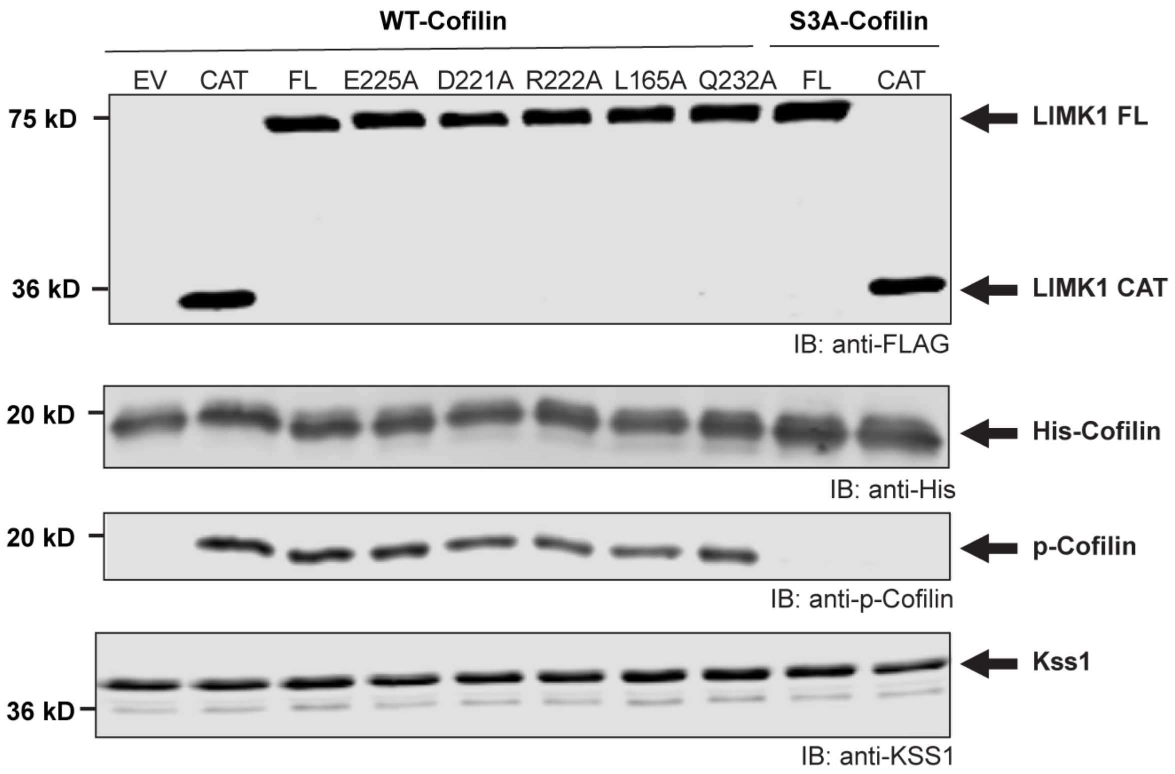

Supplementary Figure 7. LIMK1 protein expression in yeast and kinase activity assessment. Immunoblot analysis of FLAG-LIMK1 and PDZ mutants expressed in yeast Kss1 loading control, His-cofilin, and cofilin phospho-Ser3. WT indicates full length LIMK1, and CAT indicates catalytic domain. Source data are provided as a Source Data file.

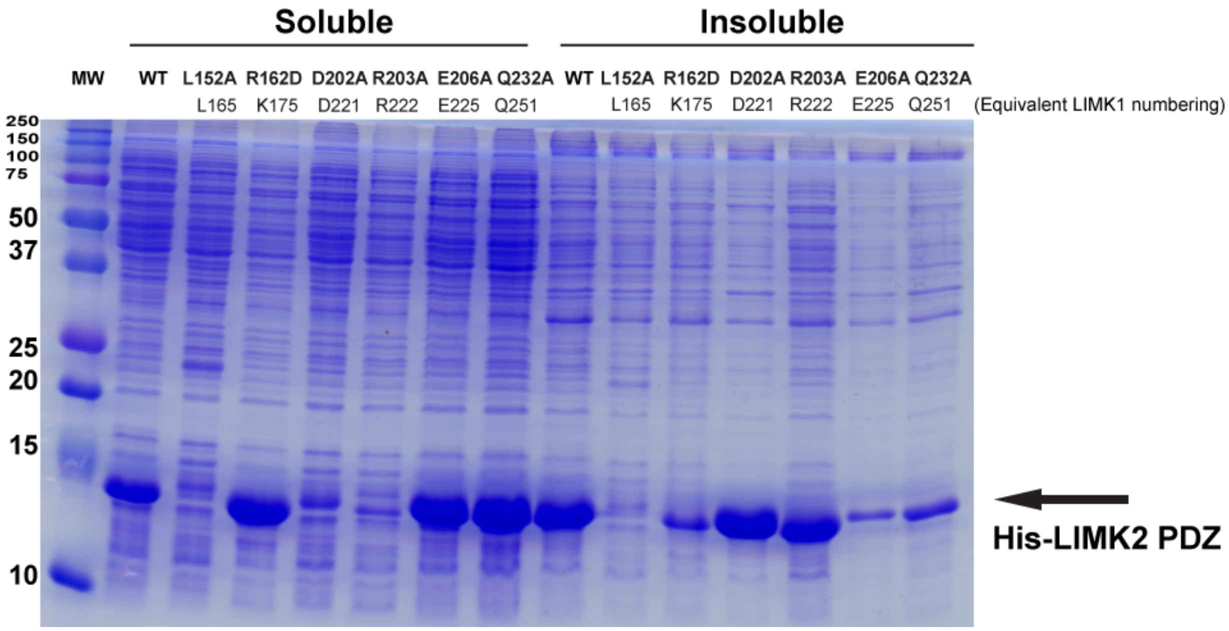

Supplementary Figure 8. Bacterial expression and solubility tests for LIMK2 PDZ domain mutants. *E. coli* lysate fractionation of the crystallized his-tagged LIMK2 PDZ domain construct, and comparison with PDZ mutants in this construct. Residue number for mutations corresponding for LIMK2 (top) and equivalent LIMK1 residue (bottom) are shown. Source data are provided as a Source Data file.

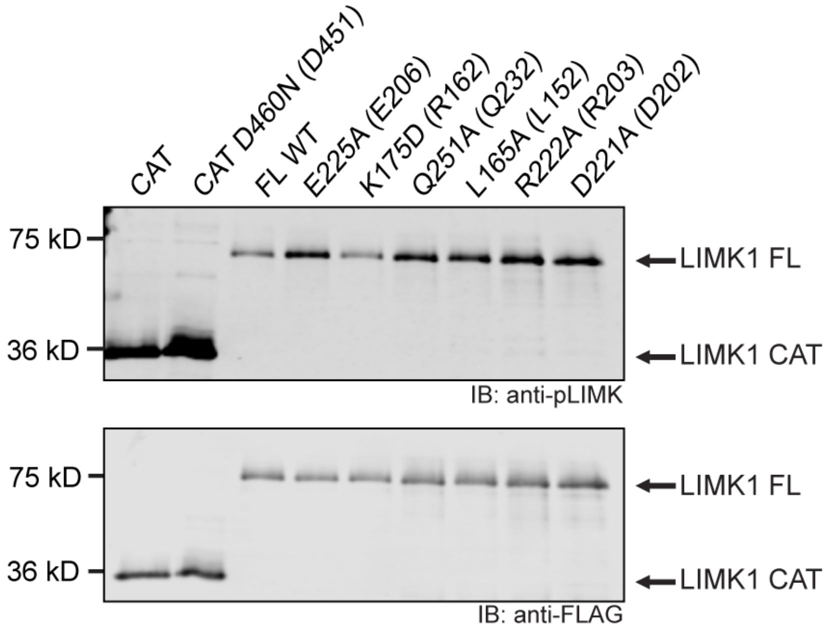

Supplementary Figure 9: Assessment of LIMK activation loop phosphorylation. Blot assessing activation loop phosphorylation in LIMK1 constructs purified from yeast. Top panel yeast lysates blotted with anti-FLAG antibody for FLAG-LIMK1 as loading control. Second panel purified yeast protein blotted with anti-phospho LIMK1. Parentheses indicate equivalent LIMK2 residue. Source data are provided as a Source Data file.

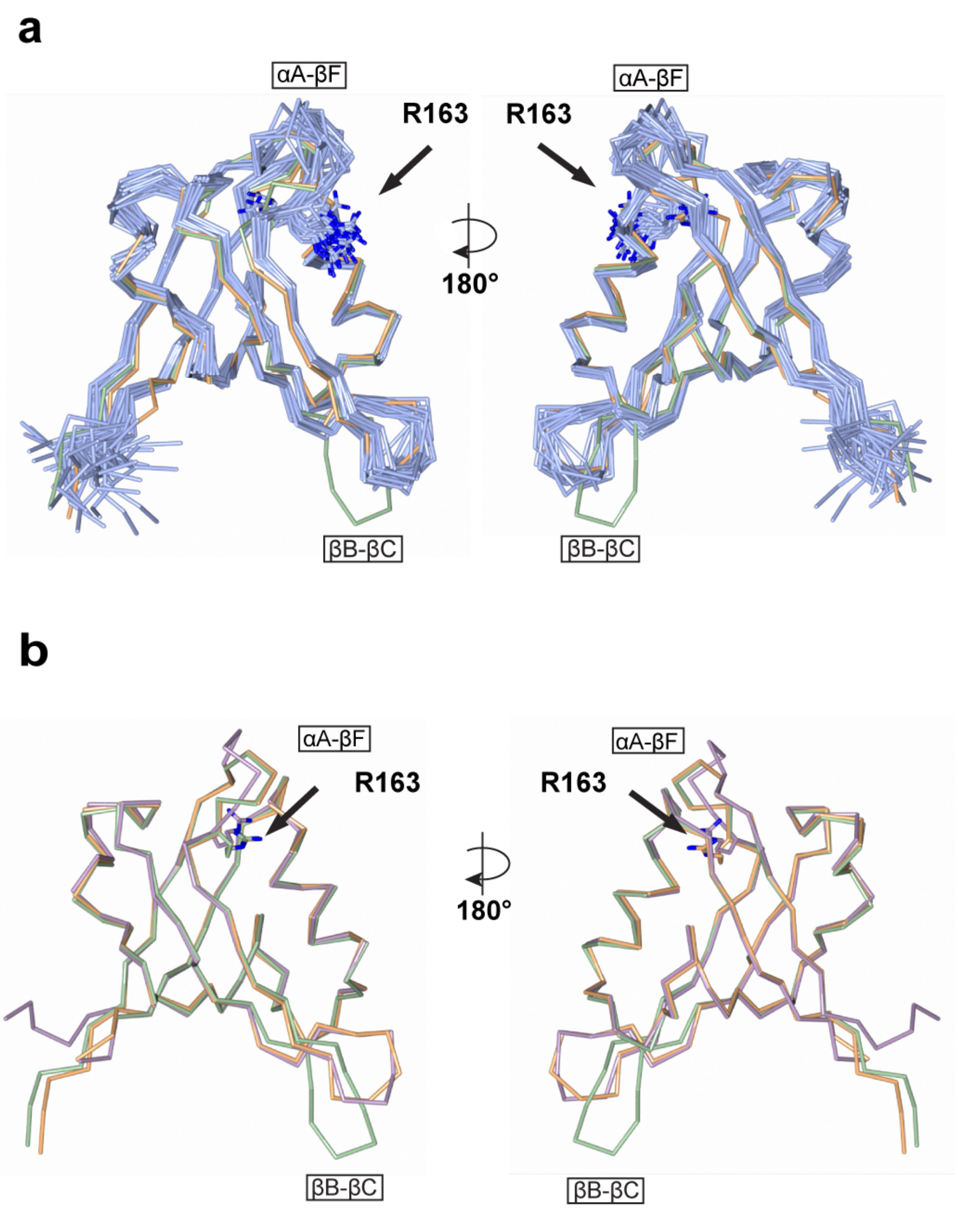

Supplementary Figure 10: Comparison of LIMK PDZ structures. a) Superposition of both conformations of the LIMK2 PDZ crystal structure (orange and green) with the 20 deposited NMR states of mouse LIMK2 PDZ domain (PDB: 2YUB; unpublished) (blue). Arg163 is indicated for the NMR structures, 17 copies are surface exposed and 3 copies point towards the core. b) Superposition of both conformations of the LIMK2 PDZ crystal structure (orange and green) with AlphaFold model of LIMK2 PDZ (pink) (LIMK2-AF-P53671-F1-model_v2.pdb).

Supplementary Table 1. Primers used for mutagenesis.

Protein Construct	Mutation	Forward primer sequence	Reverse primer sequence
pRS415-GAL-FLAG-LIMK1	L165A	5'-CGCACACCGTTACGGCGGTCAGCATTCCGG-3'	3'-CCGGAATGCTGACCGCCGTAACGGTGTGCG-5'
	D221A	5'-TCCATTCACGTTGGTGCTCGCATTCTGGAAATC-3'	3'-GATTTCCAGAATGCGAGCACCAACGTGAATGGA
	R222A	5'-ATTCACGTTGGTGATGCCATTCTGGAAATCAACGGC-3'	3'-GCCGTTGATTTCCAGAATGGCATCACCAACGTGAAT-5
	E225A	5'-GTGATCGCATTCTGGCAATCAACGGCACCCC-3'	3'-GGGGTGCCGTTGATTGCCAGAATGCGATCAC-5'
	Q251A	5'-ATGTTCCAGCGTCAGTGCCAGCAGACGGCTG-3'	3'-CAGCCGTCTGCTGGCACTGACGCTGGAACAT-5'
	K175D	5'-GCCAGCTCTCACGGTGATCGCGGCCTGTCCGTT-3'	3'-AACGGACAGGCCGCGATCACCGTGAGAGCTGGC-5'
pET HIS-hLIMK2 PDZ C173S	C173S	5'- GTGGAGAGTGCCTCCTCCAACTACG- 3'	3'- CGTAGTTGGAGGAGGCACTCTCCAC- 5'
	L152A	5'-CCCTACTCTGTCACGGCCATCTCCATGCCGGCC-3'	3'-GGCCGGCATGGAGATGGCCGTGACAGAGTAGGG-5'
	D202A	5'- GCCATCCACCCTGGGGCTCGCATCCTGG-3'	3'- CCAGGATGCGAGCCCCAGGGTGGATGGC-5';
	R203A	5'-CACCCTGGGGACGCCATCCTGGAGATCAAT-3'	3'-ATTGATCTCCAGGATGGCGTCCCCAGGGTG-5'
	E206A	5'-GACCGCATCCTGGCGATCAATGGGACCCCCGTC-3'	3'-GACGGGGGTCCCATTGATCGCCAGGATGCGGTC-5'
	Q232A	5'- CGAGCCAGACACTTGCGCTGTTGATTGAAC-3'	3'-GTTCAATCAACAGCGCAAGTGTCTGGCTCG-5'
	R162D	5'-GGCCACCACTGAAGGCGATCGGGGCTTCTCCGTG-3	3'-CACGGAGAAGCCCCGATCGCCTTCAGTGGTGGCC-5'

78. UniProt, C. UniProt: the Universal Protein Knowledgebase in 2023. *Nucleic Acids Res* (2022).

79. Gogl, G., Biri-Kovacs, B., Poti, A.L., Vadaszi, H., Szeder, B., Bodor, A., Schlosser, G., Acs, A., Turiak, L., Buday, L., Alexa, A., Nyitray, L. & Remenyi, A. Dynamic control of RSK complexes by phosphoswitch-based regulation. *FEBS J* **285**, 46-71 (2018).
