# Supplementary material for: Autoregulation of the LIM kinases by their PDZ domain
Source: Nat Commun. 2023 Dec 19;14:8441. doi: 10.1038/s41467-023-44148-4 (PMC10730565; doi:10.1038/s41467-023-44148-4)
Supplement: Supplementary file 3 — Reporting Summary [file 41467_2023_44148_MOESM3_ESM.pdf]

nature portfolio

Corresponding author(s): Titus J. Boggon

Last updated by author(s): Nov 27, 2023

Reporting Summary

Nature Portfolio wishes to improve the reproducibility of the work that we publish. This form provides structure for consistency and transparency in reporting. For further information on Nature Portfolio policies, see our Editorial Policies and the Editorial Policy Checklist.

Statistics

For all statistical analyses, confirm that the following items are present in the figure legend, table legend, main text, or Methods section.

n/a	Confirmed	
☐	☒	The exact sample size (*n*) for each experimental group/condition, given as a discrete number and unit of measurement
☐	☒	A statement on whether measurements were taken from distinct samples or whether the same sample was measured repeatedly
☐	☒	The statistical test(s) used AND whether they are one- or two-sided<br>*Only common tests should be described solely by name; describe more complex techniques in the Methods section.*
☐	☒	A description of all covariates tested
☒	☐	A description of any assumptions or corrections, such as tests of normality and adjustment for multiple comparisons
☐	☒	A full description of the statistical parameters including central tendency (e.g. means) or other basic estimates (e.g. regression coefficient) AND variation (e.g. standard deviation) or associated estimates of uncertainty (e.g. confidence intervals)
☐	☒	For null hypothesis testing, the test statistic (e.g. *F*, *t*, *r*) with confidence intervals, effect sizes, degrees of freedom and *P* value noted<br>*Give P values as exact values whenever suitable.*
☒	☐	For Bayesian analysis, information on the choice of priors and Markov chain Monte Carlo settings
☒	☐	For hierarchical and complex designs, identification of the appropriate level for tests and full reporting of outcomes
☒	☐	Estimates of effect sizes (e.g. Cohen's *d*, Pearson's *r*), indicating how they were calculated

Our web collection on statistics for biologists contains articles on many of the points above.

Software and code

Policy information about availability of computer code

Data collection	XDS version 20220110, PDB Extract 3.27, Quantity One 1D Analysis software, Image Studio Version 5.2.5
Data analysis	Phaser 2.8.3, Phenix Refine 1.19.1, Coot 0.8.9, Quantity One 1D Analysis software, GraphPad Prism Version 9.5.1, SBGrid DataBank V1.

For manuscripts utilizing custom algorithms or software that are central to the research but not yet described in published literature, software must be made available to editors and reviewers. We strongly encourage code deposition in a community repository (e.g. GitHub). See the Nature Portfolio guidelines for submitting code & software for further information.

Data

Policy information about availability of data

All manuscripts must include a data availability statement. This statement should provide the following information, where applicable:

- Accession codes, unique identifiers, or web links for publicly available datasets
- A description of any restrictions on data availability
- For clinical datasets or third party data, please ensure that the statement adheres to our policy

Coordinates and structure factors have been deposited in the Protein Data Bank under accession code 8GI4 [doi: 10.2210/pdb8GI4/pdb]. X-ray diffraction images are available online at SBGrid Data Bank [doi:10.15785/SBGRID/1010]. Previously determined structures used in our analysis were obtained from the Protein Data Bank: 3EGG [http://doi.org/10.2210/pdb3EGG/pdb] (spinophilin, PDZ), 5HEY [http://doi.org/10.2210/pdb5HEY/pdb] (disk large homolog 4 PDZ), 3K1R [http://doi.org/0.2210/pdb3K1R/pdb] (harmonin PDZ), 5G1E [http://doi.org/10.2210/pdb5G1E/pdb] (syntenin-1 PDZ). The AlphaFold model of Human LIMK2 (AF-P53671-

F1-model_v2.pdb) was obtained from the Alphafold Structure Database: https://alphafold.ebi.ac.uk/files/AF-P53671-F1-model_v2.pdb. The source data underlying Figs 4d; 5a, b; 6b, c; 7c are provided as a Source Data file. Source data are provided with this paper.

Human research participants

Policy information about [studies involving human research participants and Sex and Gender in Research.](#)

Reporting on sex and gender	N/A
Population characteristics	N/A
Recruitment	N/A
Ethics oversight	N/A

Note that full information on the approval of the study protocol must also be provided in the manuscript.

Field-specific reporting

Please select the one below that is the best fit for your research. If you are not sure, read the appropriate sections before making your selection.

☒ Life sciences ☐ Behavioural & social sciences ☐ Ecological, evolutionary & environmental sciences

For a reference copy of the document with all sections, see nature.com/documents/nr-reporting-summary-flat.pdf

Life sciences study design

All studies must disclose on these points even when the disclosure is negative.

Sample size	The sample size was chosen to ensure reproducibility. All measurements were reproducible and each measurement is plotted in the figures. At least 3 measurements were collected, which is standard in the field. For statistical analysis using the Mann-Whitney test, at least 4 samples were collected to obtain P values.
Data exclusions	No data were excluded.
Replication	All data was reproducible over independent experiments, at least three measurements per condition. Reproducibility was verified by replication in independent samples on independent days with independent measurements. The number of independent samples for each dataset is reported in the Figure legend.
Randomization	No randomization was performed since randomization is not appropriate for the experiments performed here.
Blinding	Experiments were not blinded as this was not appropriate for biochemical assays with purified proteins. The results are quantitative from endpoint assays read as Absorbance on a plate reader.

Reporting for specific materials, systems and methods

We require information from authors about some types of materials, experimental systems and methods used in many studies. Here, indicate whether each material, system or method listed is relevant to your study. If you are not sure if a list item applies to your research, read the appropriate section before selecting a response.

Materials & experimental systems

n/a	Involved in the study	
☐	☒	Antibodies
☐	☒	Eukaryotic cell lines
☒	☐	Palaeontology and archaeology
☒	☐	Animals and other organisms
☒	☐	Clinical data
☒	☐	Dual use research of concern

Methods

n/a	Involved in the study	
☒	☐	ChIP-seq
☒	☐	Flow cytometry
☒	☐	MRI-based neuroimaging

Antibodies

Antibodies used	Antibodies used for immunoblotting experiments

Antibodies used

mouse anti-FLAG antibody (Sigma, #F3165,1:5,000 dilution)
rabbit anti-KSS1 (Santa Cruz Biotechnology, # sc-6775-R , 1:5,000 dilution)
mouse anti-penta-His (Qiagen, # 34650, 1:5000)
phospho-Cofilin (phospho-Serine3) (Cell Signaling, #3311S, 1;1000)
phospho-LIMK1/LIMK2 antibody (phospho-Thr508/phospho-Thr505) (Cell Signaling, #3841S 1;1000)

Validation

Antibodies used were commercially available and were validated in multiple previous studies. Concerning antibody specificity, we refer to the supplier's websites and data sheets to find statements on specificity and dilution for the use of the antibodies.
- mouse anti-FLAG antibody (Sigma, #F3165,1:5,000 dilution); https://www.sigmaaldrich.com/US/en/product/sigma/f3165.
- rabbit anti-KSS1 (Santa Cruz Biotechnology, # sc-6775-R , 1:5,000 dilution); https://datasheets.scbt.com/sc-6775.pdf
- mouse anti-penta-His (Qiagen, #34650, 1:5000); https://www.qiagen.com/us/products/discovery-and-translational-research/protein-purification/tagged-protein-expression-purification-detection/anti-his-antibodies-bsa-free
- phospho-Cofilin (phospho-Serine3) (Cell Signaling, #3311S, 1;1000); https://www.cellsignal.com/products/primary-antibodies/phospho-cofilin-ser3-antibody/3311
- phospho-LIMK1/LIMK2 antibody (phospho-Thr508/phospho-Thr505) (Cell Signaling, #3841S 1;1000) Anti mouse antibodies: FLAG, penta-His; https://www.cellsignal.com/products/primary-antibodies/phospho-limk1-thr508-limk2-thr505-antibody/3841
0)

Eukaryotic cell lines

Policy information about cell lines and Sex and Gender in Research

Cell line source(s)

MHY8282 (a cof1Δ::kanMX/pRS316COF1) strain of S. cerevisiae provided by Mark Hochstrasser's laboratory. Strain is congenic with BY4741 (a his3Δ1 leu2Δ0 met15Δ0 ura3Δ0) strain used to make yeast gene deletion collection by Open Biosystems
Ref: Kang, H., et al. Proc Natl Acad Sci USA 111, 17821-6 (2014). PMC4273407

Authentication

Cell line was authenticated by growth on SC-Ura selective media agar plates

Mycoplasma contamination

Not checked/Not applicable

Commonly misidentified lines
(See ICLAC register)

Not applicable
